# Ab Initio Molecular Dynamics Study of Electron Excitation Effects on UO_2_ and U_3_Si

**DOI:** 10.3390/ma16216911

**Published:** 2023-10-27

**Authors:** Ruoyan Jin, Siqin Zhao, Haiyan Xiao

**Affiliations:** Yangtze Delta Region Institute (Huzhou), University of Electronic Science and Technology of China, Huzhou 313001, China; jinruoyan@std.uestc.edu.cn (R.J.); aa1034753474@163.com (S.Z.)

**Keywords:** nuclear fuels, ab initio molecular dynamics, electron excitation, structural amorphization

## Abstract

In this study, an ab initio molecular dynamics method is employed to investigate how the microstructures of UO_2_ and U_3_Si evolve under electron excitation. It is found that the U_3_Si is more resistant to electron excitation than UO_2_ at room temperature. UO_2_ undergoes a crystalline-to-amorphous structural transition with an electronic excitation concentration of 3.6%, whereas U_3_Si maintains a crystalline structure until an electronic excitation concentration reaches up to 6%. Such discrepancy is mainly due to their different electronic structures. For insulator UO_2_, once valence U *5f* electrons receive enough energy, they are excited to the conduction bands, which induces charge redistribution. Anion disordering is then driven by cation disordering, eventually resulting in structural amorphization. As for metallic U_3_Si, the U 5*f* electrons are relatively more difficult to excite, and the electron excitation leads to cation disordering, which eventually drives the crystalline-to-amorphous phase transition. This study reveals that U_3_Si is more resistant to electron excitation than UO_2_ under an irradiation environment, which may advance the understanding of related experimental and theoretical investigations to design radiation-resistant nuclear fuel uranium materials.

## 1. Introduction

As one of the significant sources of carbon-free energy, nuclear energy has been used on a massive scale. The selection of nuclear fuel is key to deciding the safety of nuclear energy technology, as well as its stability and economic efficiency [1]. In the past decades, uranium has been used as nuclear fuel in fission reactors, and some uranium complexes have been investigated for long-term nuclear waste disposal and the storage of highly radioactive waste materials [2,3]. Throughout the world, UO_2_ has been employed in a large number of commercial light water reactors because of its superior properties, such as high melting point (3147 ± 20 K) [4,5,6], good oxidation resistance, and chemical compatibility [1,7]. However, the poor thermal conductivity of UO_2_ (2∼6 Wm^−1^K^−1^) in the temperature range of 273∼1673 K [8] can cause a large temperature gradient during operation [1,7]. Therefore, extensive studies have been conducted to search for alternative fuels with better thermal performance in reactors [7,9,10]. In addition to UO_2_, other types of nuclear fuels, including mixed oxides ((U,Pu)O_2_, (U,Th)O_2_, (Pu,Th)O_2_) [11,12], alloys (U-Al, U-Mo, U-ZrH) [13], UC [14], UN [15], and uranium silicides such as U_3_Si [16] and U_3_Si_2_ [17,18], have been used or proposed for different reactors. Among these nuclear fuels, the U_3_Si_2_ and U_3_Si, due to their high uranium density [19] and large thermal conductivity [20], are considered potential accident-tolerant fuels. The U_3_Si has a thermal conductivity of approximately 15∼25 Wm^−1^K^1^ under 300∼1100 K [21] and a maximum uranium loading of 14.6 g-U/cm^3^, which is much higher than that of UO_2_ (9.7 g-U/cm^3^) [16,22]. To date, the U_3_Si is a potential nuclear fuel in low-temperature and low-power reactors such as the Miniature Neutron Source reactors [19,23,24].

During the past decades, researchers have striven to investigate the response of microstructural change in nuclear materials under ion, electron, and pulsed laser irradiation [25,26]. Barthe et al. irradiated sintered UO2 disks with electrons and α particles at different fluences [27]. The results showed the formation of U-related vacancy defects after a 2.5 MeV electron irradiation, whereas no defects were detected for irradiation at 1 MeV. Experimentally, Onofri et al. characterized the microstructural evolution of poly-crystalline UO_2_ under 4 MeV Au and 390 keV Xe ions irradiation with fluences of 0.5 × 10^15^~1.0 × 10^15^ ions/cm^2^ and found several dislocation loops and dislocation lines [24]. Miao et al. studied the high-burnup structure in UO_2_ under 84 MeV Xe ion irradiation and found that radiation-induced dislocations result in grain polygonization [28]. In addition, Miao et al. investigated the response of U_3_Si_2_ to 84 MeV Xe ion irradiation at 600 °C and found that the U_3_Si_2_ is strongly resistant to radiation-induced amorphization [29]. Theoretically, Owen studied amorphous UO_2_ systems using classical molecular dynamics methods and reported that the amorphous structure of UO_2_, i.e., oxygen ions, are coordinated with 3.65 uranium ions and uranium ions are coordinated with 7.31 oxygen ions [6]. Moreover, ab initio molecular dynamics (AIMD) computer simulations of low-energy recoil events in UO_2_ and ThO_2_ were carried out by Xiao et al., who determined the threshold displacement energies and revealed a number of novel point defects [30,31].

Ion or laser irradiation can substantially deposit energy in solids via elastic and inelastic collisions, as described in [32,33,34], which induces strong electron excitation and ionization [33,35]. In the literature, researchers have demonstrated that electron excitation can induce crystalline-to-amorphous structural transitions and influence material properties significantly. For example, Li et al. studied the response of Ge-Sb-Te alloys to laser irradiation by employing an AIMD method and found that electron excitation results in the structural amorphization of the alloy [36]. The amorphization of crystalline SiO_2_ induced by an electron beam in transmission electron microscopes has been reported by a number of researchers [37,38,39]. Furthermore, Xiao et al. carried out AIMD simulations on a series of titanate pyrochlores under electron irradiation and found that a crystalline-to-amorphous structural transition is induced by electron excitation in these pyrochlores [40]. Zhao et al. performed finite-temperature density functional theory and AIMD simulations on SrTiO_3_ and reported that there is a charge redistribution in Ti-O bonds and that the SrTiO_3_ undergoes a phase transition from crystalline to an amorphous state under electron excitation [33]. Thus far, the microstructural evolution of UO_2_ and U_3_Si under electron excitation still remains unclear. In this work, the structural evolution of UO_2_ and U_3_Si under electron excitation is investigated by employing the AIMD method, and the possible origin of their different response behaviors is explored. It is revealed that the U_3_Si is more resistant to electron excitation than the insulating UO_2_ due to its metallic character, suggesting that the U_3_Si has excellent structural stability under electron and laser irradiation.

## 2. Computational Methods

**Figure 1:**
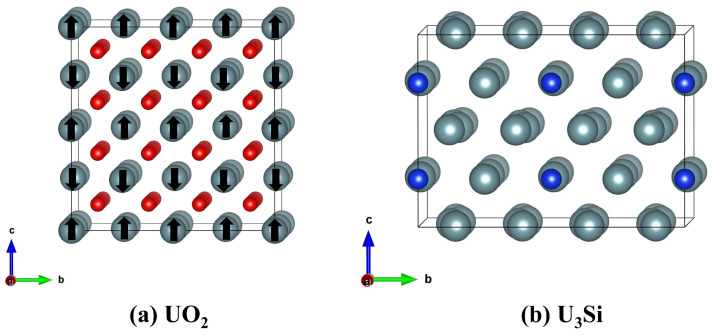
Schematic view of the geometrical structures of (**a**) UO_2_ and (**b**) U_3_Si. The grey, red, and blue spheres represent the U, O, and Si atoms, respectively. For UO_2_, anti-ferromagnetic ordering is illustrated, and the “↑” and “↓” signs on U atoms denote spin up and spin down, respectively.

Because of the existence of U 5*f* electrons in UO_2_ and U_3_Si, strong correlation effects cannot be ignored [41]. To correct the on-site Coulomb interaction between the U 5*f* electrons, the Hubbard U correction proposed by Dudarev et al. [42] is utilized in this work. In this study, the responses of UO_2_ and U_3_Si to electron excitation are simulated by using the AIMD + U (ab initio molecular dynamics plus Hubbard U correction) method, as implemented in the Vienna Ab Initio Simulation Package (VASP) [43]. The effective Hubbard parameter $U_{\mathrm{eff}}=U-J$ is employed to be 4.0 eV [44] for UO_2_ and 1.5 eV [45] for U_3_Si. The interactions between ions and electrons are described by the projector-augmented wave (PAW) pseudopotential, as described in [46,47] and the exchange–correlation potential between electrons is described by the Ceperly Alder parameterization under local density approximation (LDA) [48]. With spin-polarized effects taken into account, the cut-off energy for the plane-wave basis is set to 400 eV. A 1 × 1 × 1 K-point sampling in the Brillouin zone created by the Monkhorst-Pack [49] technique is used in AIMD calculations. Uranium dioxide (UO_2_) is of a fluorite structure in which the cations are located in a face-centered cubic (fcc) sublattice and the anions are in a cubic sublattice [30]. The U_3_Si crystal is of a tetragonal structure with the space group I4/mcm [50]. The UO_2_ has 12 atoms in the unit cell and the U_3_Si has 16 atoms in the unit cell. In the AIMD simulation, a 2 × 2 × 2 (96 atoms) and a 2 × 2 × 1 (64 atoms) supercell are employed for UO_2_ and U_3_Si, respectively, and periodic boundary conditions are applied along three directions. Figure 1 shows a schematic view of the geometrical structures of UO_2_ and U_3_Si. For UO_2_, anti-ferromagnetic ordering is considered, and the magnetic moment is determined to be 2.06 µB, which agrees well with the experimental value of 2 µB [51]. Electron excitation can be achieved by removing several electrons from the valence band states during the AIMD simulation, and the charge loss due to electron removal is compensated by a jellium background [36]. The electronic excitation concentration is defined as the ratio of the number of excited electrons to the total number of electrons. For a 2 × 2 × 2 supercell of the UO_2_ crystal, the total number of electrons is 3456, and the number of removed electrons ranges from 83 to 208, corresponding to an excitation concentration of 2.4~6%. For a 2 × 2 × 1 supercell of U_3_Si crystal, the total number of electrons is 4640, and the number of removed electrons ranges from 111 to 278, corresponding to an excitation concentration of 2.4~6%. The intensity of the generated electron-hole pairs can be calculated as $N_{e-h}=\frac{\left(1-R\right)\times \alpha_{eff}\times F}{\hbar \omega_{0}}$, where *F* and $\omega_{0}$ represent the laser fluence and frequency, respectively, and *R* and $\alpha_{eff}$ represent the sample’s reflectivity and effective absorption coefficient, respectively [52]. Under laser beam irradiation, employing volume = 1.31 × 10^−21^ cm^3^, $\hbar \omega_{0\;}$ = 5 eV, R (248 nm) = 22% [53], and $\alpha_{eff}$ (248 nm) = 0.43 × 10^5^ cm^−1^, the predicted laser fluence at 248 nm for 3.6% excitation in UO_2_ is 2.27 × 10^3^ mJ/cm^2^. The AIMD simulation is conducted with a time step of 1.5 fs and a canonical ensemble, and the Nosé–Hoover thermostat [54] is employed to control the temperature.

## 3. Results and Discussion

**Figure 2:**
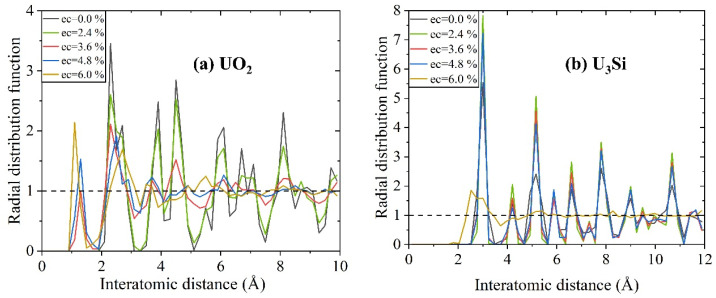
The radial distribution functions of (**a**) UO_2_ and (**b**) U_3_Si as a function of electronic excitation concentration at 300 K.

The RDF defines variations in the surrounding matter density as a function of distance from a point, as well as the frequency with which specific distances occur [55], which is obtained in the simulation model by counting the number of atom pairs separated by particular distances. Figure 2 illustrates the RDFs of UO_2_ and U_3_Si with and without electron excitation at a temperature of 300 K. When a material is crystalline, its structure is ordered at both short-range and long-range distances; when the material is amorphous, its structure is ordered at short-range distances and disordered at long-range distances. The strong and weak RDFs (with peak values approaching 1) correspond to structural ordering and disordering, respectively. Figure 2a presents the radial distribution functions of UO_2_ and U_3_Si as a function of the electronic excitation concentration at 300 K. For UO_2_, it is noted that its structure remains ordered at both short-range and long-range distances under electronic excitation concentrations of 0% and 2.4%, as indicated by the strong peaks in the whole considered interatomic distance range; however, as the electronic excitation concentration increases to 3.6%, structural short-range ordering and long-range disordering are observed, i.e., a crystalline-to-amorphous phase transformation is induced. A similar phenomenon has been observed in SrTiO_3_, titanate pyrochlores, and nickel oxide [33,40,56] for which electron excitation also causes a crystalline-to-amorphous phase transformation. In particular, new peaks appear at 1.17~1.3 Å, which can be attributed to the formation of O_2_ molecules. In the case of U_3_Si, it is noticeable that the situation is somewhat different. U_3_Si is shown to be strongly tolerant to electron excitation and maintains a crystalline structure under electronic excitation concentrations of 0.0%, 2.4%, 3.6%, and 4.8%, as indicated by the strong RDF peaks in the whole interatomic distance range in Figure 2b. When the electronic excitation concentration is as high as 6.0%, a crystalline-to-amorphous phase transformation occurs. Obviously, U_3_Si is more resistant to electron excitation than UO_2_.

**Figure 3:**
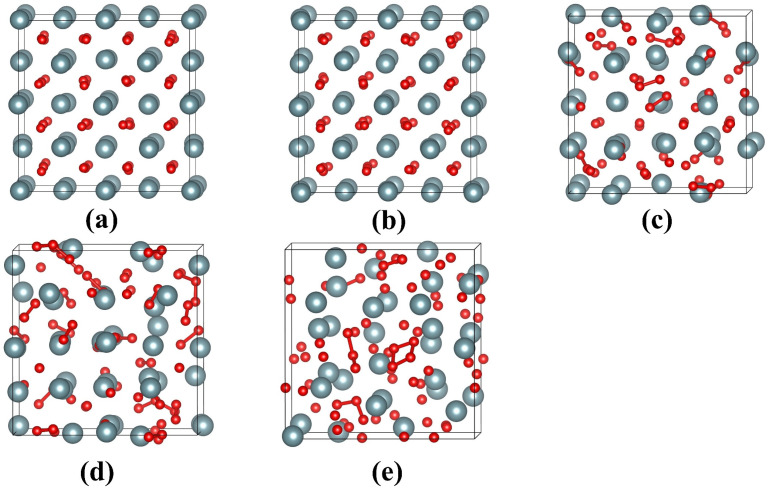
Equilibrium geometrical structures of UO_2_ with electronic excitation concentrations of (**a**) 0.0%; (**b**) 2.4%; (**c**) 3.6%; (**d**) 4.8%; (**e**) 6.0% at 300 K. The grey and red spheres represent U and O, respectively. The <O-O> chemical bonds between 1.17 and 1.3 Å are represented by red sticks.

**Figure 4:**
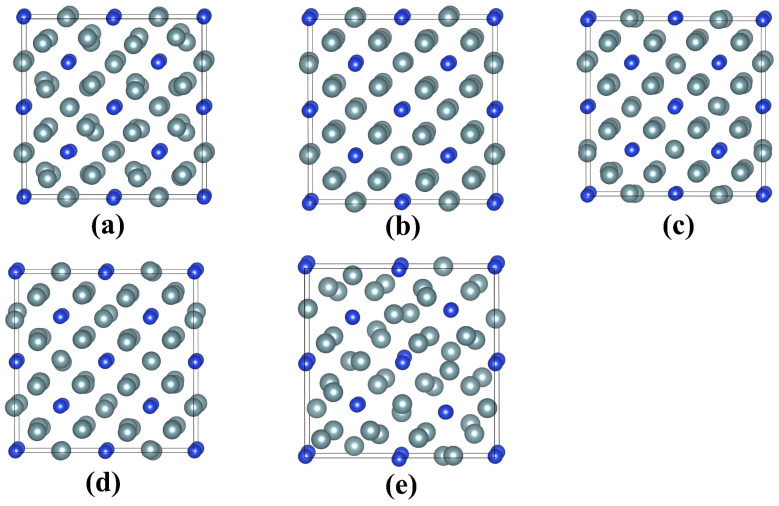
Equilibrium geometrical structures of U_3_Si with electronic excitation concentrations of (**a**) 0.0%; (**b**) 2.4%; (**c**) 3.6%; (**d**) 4.8%; (**e**) 6.0% at 300 K. The grey and blue spheres represent U and Si, respectively.

Figure 3 illustrates the geometrical configurations of UO_2_ after electron excitation at different electronic excitation concentrations. It is shown that the UO_2_ remains in a crystalline structure when no electrons are excited. For an electronic excitation concentration of 2.4%, there is a slight lattice disordering for anions, and the cations maintain the lattice ordering well. When the electronic excitation concentration reaches 3.6%, the structure of UO_2_ changes significantly. In particular, the anions are dramatically disordered, and O_2_-like molecules are formed. The <O-O> distances are determined to be approximately 1.13 Å, which is close to the bond length of 1.24 Å for O_2_ [40]. As the electronic excitation concentration further increases to 4.8% and 6.0%, more and more O_2_-like molecules are formed. Moreover, cation disordering has become increasingly significant. Obviously, during the electron excitation process, anions are first disordered, which drives cation disordering and eventual structural amorphization. Such an amorphization mechanism is found to be similar to that of titanate pyrochlores [40]. In comparison to UO_2_, electron excitation with 2.4~6.0% concentration has a more limited influence on the geometrical structure of U_3_Si. As can be seen from Figure 4b–d, for electronic excitation concentrations of 2.4%, 3.6%, and 4.8%, the structures of U_3_Si are well ordered; in the case of 6.0% electronic excitation concentration, it is noted that lattice disordering occurs on U atoms rather than Si atoms (see Figure 4e), suggesting that the structural amorphization in U_3_Si is driven by cation disordering instead of anion disordering. This is different from the amorphization mechanism of UO_2_. Experimentally, the microstructural evolution in UO_2_ and U_3_Si under electron excitation has not been investigated yet, since they are radioactive materials and direct experimental study is not easy. However, electron excitation-induced structural amorphization has been experimentally observed in alloys, semiconductors, and ceramics [38,57,58,59].

**Figure 5:**
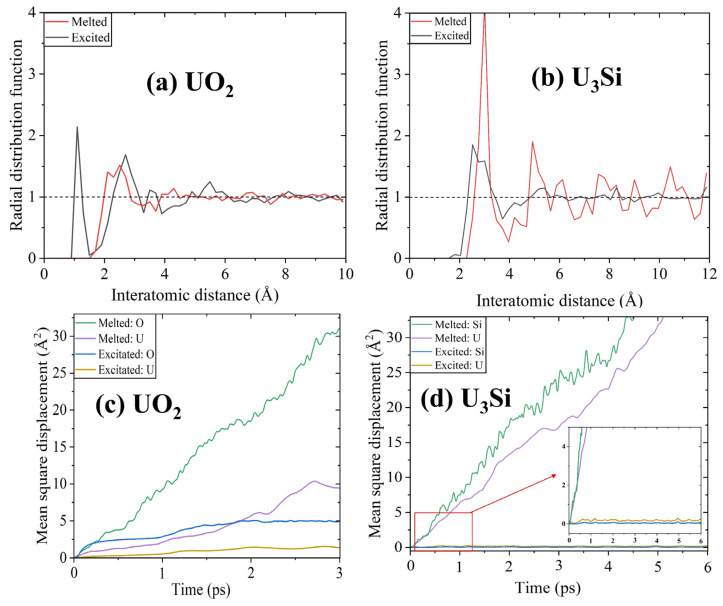
(**a**,**b**) The radial distribution functions and (**c**,**d**) mean square displacements of UO_2_ and U_3_Si. The melted UO_2_ and U_3_Si are simulated at a temperature of 5000 K above their melting points. The excited UO_2_ and U_3_Si are simulated with an electronic excitation concentration of 6.0%.

To evaluate if the structural amorphization is a solid–liquid transition, an AIMD approach is utilized to simulate the structural evolution of UO_2_ and U_3_Si at 5000 K, which is much higher than their respective melting temperatures of 3147 ± 20 K [4,5] and ~1258 K [60]. Figure 5a compares the RDFs of melted and excited UO_2_ with an electronic excitation concentration of 6.0%. As shown in Figure 5a, the RDF peak located at an interatomic distance of ~1.13 Å for excited UO_2_ disappears for melted UO_2_, implying that no O_2_-like molecules are formed during the melting process. In the case of U_3_Si (see Figure 5b)**,** the peak intensity of RDF at short-range distances for melted U_3_Si is much stronger than that for excited U_3_Si, indicating strengthened short-range ordering. Obviously, the structural amorphization induced by high-temperature melting is different from that induced by electron excitation. Figure 5c,d compares the mean square displacements of cations and anions in melted and excited UO_2_ and U_3_Si with a 6% electronic excitation concentration. As shown in Figure 5c, with a simulation time of 3 ps, the displacements of O and U atoms in melted UO_2_ are approximately six and ten times larger than those of excited UO_2_, respectively. As for U_3_Si, after a simulation time of 6 ps, the displacements of U and Si atoms in melted U_3_Si are over thirty times larger than those in excited U_3_Si, as illustrated in Figure 5d. These results suggest that the electron excitation-induced phase transition in UO_2_ and U_3_Si is different from the thermal melting-induced phase transition, and the former is a solid–solid transition rather than a solid–liquid transition. In the literature, a similar phenomenon is reported for titanate pyrochlores [40] and In_2_Se_3_ nanowires [61].

**Figure 6:**
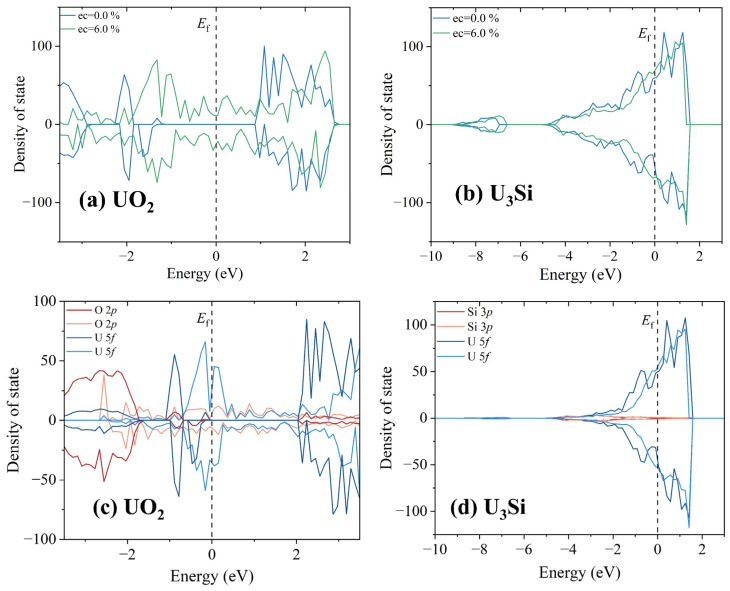
The total density of state distributions for (**a**) UO_2_ and (**b**) U_3_Si without and with an electronic excitation concentration of 6.0%; (**c**) partial density of state distributions projected on 2*p* orbitals of O atom and 5*f* orbitals of U atom of UO_2_ without and with an electronic excitation concentration of 6.0%; (**d**) partial density of state distributions projected on 3*p* orbitals of Si atom and 5*f* orbitals of U atom of U_3_Si without and with an electronic excitation concentration of 6.0%.

To explore the origin of the discrepancy in microstructural evolution under electron excitation between UO_2_ and U_3_Si, the total and projected density of state distributions of UO_2_ and U_3_Si with and without an electronic excitation concentration of 6.0% are illustrated in Figure 6. The Fermi level is set to be 0 eV. For ground state UO_2_, the band gap is determined to be 1.89 eV without spin–orbital coupling compared with the experimental value of ~2.1 eV [62]. In the case of ground state U_3_Si, electrons are distributed on the Fermi level, indicative of metallic character, which agrees well with the experimental results [24]. Clearly, the electronic structure of ground state UO_2_ is very different from that of ground state U_3_Si, which may result in different responses of UO_2_ and U_3_Si to electron excitation. For UO_2_, the valence band maximum is mainly contributed by U *5f* orbitals, indicating that the valence electrons located at U *5f* orbitals are relatively more easily excited. When the number of excited electrons is large enough, the excitation of U *5f* electrons induces charge redistribution, resulting in cation disordering and the generation of O_2_-like molecules. As for U_3_Si, the U 5*f* electrons dominate the Fermi level. Therefore, the structural amorphization in U_3_Si is also driven by cation disordering. Since the electron work function of the U element is as high as 3.63 eV, as described in [63,64], the electrons in U_3_Si are more difficult excite than those in UO_2_. Consequently, the U_3_Si is more resistant to electron excitation than the UO_2_.

## 4. Conclusions

In summary, an AIMD simulation method is employed to investigate the response behaviors of UO_2_ and U_3_Si to electron excitation at room temperature. It is found that a crystalline-to-amorphous phase transition can be induced in UO_2_ with an electronic excitation concentration of 3.6%, whereas in U_3_Si, the threshold electronic excitation concentration for structural amorphization is as high as 6.0%. This structural amorphization induced by electron excitation is a solid–solid transition rather than a solid–liquid transition. The different electronic structures of ground-state UO_2_ and U_3_Si might result in different responses to electron excitation. The UO_2_ is an insulator, for which the U *5f* valence electrons are relatively more easily excited, which drives anion disordering and eventually structural amorphization. U_3_Si exhibits a metallic character, and the U 5*f* electrons dominate the Fermi level. Electron excitation causes cation disordering, which leads to structural amorphization. This study reveals that U_3_Si undergoes crystalline-to-amorphous structural transitions less easily than UO_2_ when electronic excitation occurs, suggesting that U_3_Si has better potential under electron or laser irradiation as a nuclear fuel than UO_2_.

## Data Availability

The raw/processed data required to reproduce these findings cannot be shared at this time as the data also form part of an ongoing study.
